# Lateral medullary syndrome in a resources limited hospital: A rare clinical anatomical variation of ischemic stroke

**DOI:** 10.1002/ccr3.8976

**Published:** 2024-05-26

**Authors:** Ahmed Alsiddig Ebraheem, Mumen Abdalazim Dafallah, Khawla Abdelmonem Yousef Mohamamed

**Affiliations:** ^1^ Internal Medicine Department, Faculty of Medicine University of Gezira Wad Medani Gezira State Sudan; ^2^ Internal Medicine Department Sudan Medical Specialization Board Wad Medani Gezira State Sudan; ^3^ Faculty of Medicine University of Gezira Wad Medani Gezira State Sudan

**Keywords:** lateral medullary syndrome, resources limited hospital, stroke, Sudan, Wallenberg syndrome

## Abstract

**Key Clinical Message:**

Although it is rare, physicians should be familiar with the presentation of lateral medullary syndrome (LMS). Urgent neuroimaging is crucial to distinguish LMS from other causes of stroke. The majority experience significant improvement within months.

**Abstract:**

Lateral medullary syndrome is a rare type of stroke resulting from a vascular event in the lateral part of the medulla oblongata. Loss of pain and temperature in the ipsilateral side of the face, and contralateral side of the body along with ipsilateral ataxia, vertigo, nystagmus, dysphagia, and hiccups are the hallmark clinical presentation. We reported a case of a 51‐year‐old male with a long history of smoking and newly discovered hypertension who presented complaining of vomiting, regurgitation, and hiccups for 1 month; tingling and numbness sensation in the left side of the face and the right side of the body, and unsteady gait for 2 weeks. Neurological examinations revealed left‐sided ptosis and miosis, diminished sensation of the three divisions of the trigeminal nerve, deviated uvula to the right side, absent gag reflex, and intention tremors. The patient received the appropriate treatment; showed a good recovery with his symptoms, was able to walk unsteady, and was discharged after 10 days in a good condition.

## 1 INTRODUCTION

Lateral medullary syndrome (LMS), also called Wallenberg syndrome or posterior inferior cerebellar artery (PICA) syndrome, results from a vascular event in the lateral part of the medulla oblongata.^1^ The vertebral artery and its largest branch; the PICA are the most common arteries involved in LMS.^2^ Various etiologies have been linked with developing LMS; atherosclerosis, hypertension, cardioembolism, and small vessel diseases are the most important.^1^, ^3^ Symptoms that are usually developed are vertigo, dizziness, nystagmus, ataxia, nausea, vomiting, dysphagia, and hiccups.^2^, ^4^ Involvement of vestibular nuclei, vestibular–cerebellar connections, spinothalamic tract, trigeminal nerve, vestibular nucleus, and descending sympathetic tract are the key pathophysiological processes in the development of LMS.^5^, ^6^

Here, we reported a case of LMS in a resourced limited hospital in central Sudan. To the best of our knowledge, this is the first reported case of LMS in a Sudanese patient.

## 2 CASE HISTORY/EXAMINATION

A 51‐year‐old Sudanese male worker with a clear medical background presented to the emergency department with vomiting and regurgitation. History revealed that the condition started 1 month ago with repeated attacks of vomiting, about three to four times per day, in large amounts, containing food particles, and associated with nausea, early satiety, and epigastric pain. Three days later, he developed frequent attacks of hiccups, precipitated mainly by large meals, accompanied by regurgitation, and not associated with other neurological symptoms. Later on, he complained of gradually blurred vision, bilateral (mainly in the left eye), not associated with pain, discharge, double vision, or headache, and not preceded by trauma. One week later, he developed a cough associated with hoarseness of voice, excessive sneezing, and dysphagia. The dysphagia was mainly solid, intermittent, with difficulty in initiation swallowing and sensation of food sticking in the throat, and not associated with odynophagia. Two weeks later, he complained of tingling and numbness sensation in the left side of his face and the right side of the trunk and extremities. He denied any motor weakness. He also felt dizzy when tried to get out of his bed, was unable to balance himself, and had a tendency to fall toward his left side. In addition, he complained of hand tremor, bilaterally (mainly in the left hand), noticed mainly when tried to initiate movement, has no relation to rest, and is not associated with any abnormal or slowness of movement. To measure the degree of disability, the Modified Rankin Scale (MRS) was applied; our patient had moderate severe disability; unable to walk and attend to bodily needs without assistance; and therefore scored 4 on the score. No relevant past medical history was present, and he was not on regular medications. No family history of a similar condition. Importantly, he had a 25‐year smoking history.

His blood pressure is 170/110 mmhg, pulse rate 82 bpm, respiratory rate 26 c/pm, and a febrile. The cardiovascular, respiratory, and abdominal examinations were unremarkable, and his higher functions, skull, and neck examinations were normal. Cranial nerve examinations revealed left‐sided ptosis and constricted pupil (miosis); findings consistent with partial Horner syndrome. Pinprick sensation for the three divisions of the trigeminal nerve was diminished, and the corneal and jaw reflexes were intact. The uvula deviated to the right side, and the gag reflex was absent. The patient's visual field was normal, with full extraocular muscle movement; however, he has a nystagmus. His facial expression was normal bilaterally. Both superficial and deep reflexes were normal. Coordination assessment through finger to finger to finger to nose test and supination and pronation (dysdiadochokinesia) were limited in the left hand; importantly, he has intention tremors noticed mainly in the left hand. Heel knee chin test/heel‐to‐shin test were normal. In addition, he was unable to balance himself when he tried to stand from the sitting position and had truncal ataxia.

### 2.1 Methods (differential diagnosis, investigations, and treatment)

**FIGURE 1:**
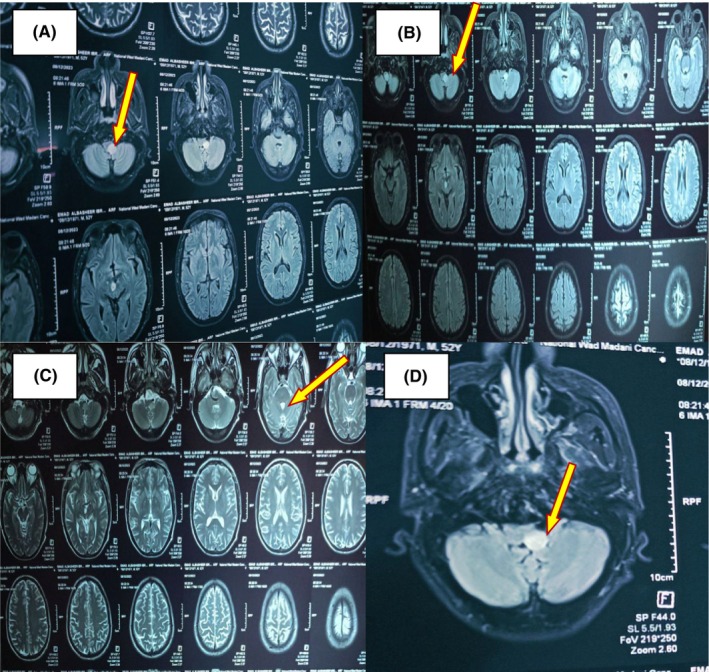
(A–D) Brain MRI showed 1.4 × 1.3 cm low T1 and high T2 right lateral cerebello‐pontomedullary ischemic lesion exert homogenous post‐contrast enhancement.

Routine blood tests, carotid Doppler scan, electrocardiogram, echocardiography, and brain CT were normal. Upper gastrointestinal endoscopy showed weak right vocal cord movement. Brain MRI revealed 1.4 × 1.3 cm low T1 and high T2 right lateral cerebello‐pontomedullary ischemic lesion exert homogenous post‐contrast enhancement Figure 1A–D.

### 2.2 Diagnostic challenges and study limitations

MRI with diffusion‐weighted imaging (DWI) and magnetic resonance angiography (MRA) were not done due to the unavailability in our resources limited hospital. Thus, we made the diagnosis of LMS on clinical background with the aid of findings in the brain MRI.

Emergency treatment with tissue plasminogen activator was not initiated due to the delayed presentation. However, he was managed with dual‐anti‐platelet therapy (aspirin 100 mg once daily and clopidogrel 75 mg once daily) and atorvastatin 40 mg once daily. Vomiting was controlled via metoclopramide intra muscular once daily, along with ondansetron 8 mg twice daily. Carbamazepine 20 mg once daily was given for numbness, and the blood pressure was controlled by amilodipine 10 mg once daily and valsartan 160 mg once daily.

## 3 CONCLUSION AND RESULTS (OUTCOME AND FOLLOW‐UP)

The patient showed a good recovery with his symptoms (vomiting, dysphagia, hand tremors, and numbness) and was able to walk unsteady. He was discharged after 10 days in good condition. On discharge, he scored 1 on the MRS (no significant disability despite symptoms; able to carry out all usual duties and activities); Table 1.

**TABLE 1:** The Modified Rankin Scale (MRS) at presentation and discharge.

Score	MRS	
0	No symptoms at all	Our patient's Scale at the time of presentation = 4
1	No significant disability despite symptoms; able to carry out all usual duties and activities	
2	Slight disability; unable to carry out all previous activities, but able to look after own affairs without assistance	
3	Moderate disability; requiring some help, but able to walk without assistance	Our patient's scale on discharge = 1
4	Moderately severe disability; unable to walk and attend to bodily needs without assistance	
5	Severe disability: bedridden, incontinent, and requiring constant nursing care and attention	
6	Dead	

## 4 DISCUSSION

LMS; also called Wallenberg syndrome; relies on the scientist Adolf Wallenberg who first described infarction in the lateral part of the medulla oblongata in 1895 as the primary cause of this condition.^7^ Atherothrombotic occlusion of the vertebral artery and the occlusion of PICA are the main vascular events encountered in LMS. Hypertension, diabetes, smoking, connective tissue diseases, and vertebral artery dissection were linked with developing LMS. Atherosclerotic risk factors are usually linked with developing LMS in elderly patients, whereas vertebral artery dissection is reported in younger patients.^5^, ^6^, ^8^ LMS is classified as the most frequent type of posterior ischemic stroke with nearly about 60,000 new cases diagnosed annually in the United States. The condition usually affects adults in the sixth decade of life and tends to affect males more than females.^9^ Thus, a typical patient is an elderly male with vascular risk factors. Importantly, our patient is a 51‐year‐old male with a 25‐year smoking history, and he has a newly discovered hypertension.

Careful history and detailed neurological examination are crucial for diagnosis. The triad of ipsilateral ataxia, horner's syndrome, and contralateral hypalgesia are the hallmark clinical features of LMS. Vertigo, nausea, and ataxia are usually developed early in the course of the disease, as in our patient; nevertheless, different combinations and presentations could be elicited in LMS.^5^ Table 2 describes the tracts and pathways involved in LMS.

**TABLE 2:** The involved tracts and pathways in lateral medullary syndrome.

The involved area/tract/pathway on the side of the lesion	The presenting symptoms/signs	Our patient's findings
Sympathetic fibers	Miosis, ptosis, and anhydrosis (Horner syndrome)	Miosis and ptosis
Inferior cerebellar hemisphere, spinocerebellar fibers, and inferior cerebellar peduncle	Ipsilateral ataxia, dysmetria, past pointing, intention tremor, and dysdiadochokinesia	Ipsilateral ataxia, dysmetria, past pointing, intention tremor, and dysdiadochokinesia
Descending trigeminal tract	Loss of pain and temperature in the face	Loss of pain and temperature in the face
Inferior vestibular nucleus and pathways	Vertigo with nystagmus	Vertigo with nystagmus
Different nuclei and fibers of the IX and X nerves	Dysphonia, dysarthria, and dysphagia	Dysphagia
Involvement of nucleus tractus solitarius	Impaired taste sensation	Not involved
On the contralateral side		
Spinothalamic tract	Loss of pain and temperature in the trunk and limbs	Loss of pain and temperature in the trunk and limbs
Ccorticospinal fibers	No or only minimal weakness of the contralateral side	Not involved

The diagnosis of LMS is usually suspected from the presenting history and the clinical examination. All patients with suspected LMS should be tested by the three‐step‐oculomotor examination head‐impulsive‐nystagmus‐test‐of‐skew (HINTS). This test showed a high validity in determining the presence of LMS in patients presenting with symptoms suggesting acute vestibular syndrome.^6^ One study concluded that the HINTS detected the presence of stroke with 100% sensitivity and 96% specificity and, importantly, detected all patients with LMS.^10^ Importantly, the maneuver revealed a direction‐changing nystagmus in our patient. Carotid Doppler scan, electrocardiogram, and echocardiography should be done to exclude any cardiac event or possible vascular stenosis. It is important to note that stroke could be the initial presentation of hematological disorders, for example, essential thrombocythaemia, polycythemia vera, smoker's polycythemia, thrombotic thrombocytopenic purpura, sickle‐cell disease, and others. However, it is more common to encounter younger patients with abnormal hematological or coagulation screening.^11^

Brain CT scan was normal in our patient; brain CT usually has a limited visualization of the posterior fossa; thus, its usefulness in detecting LMS is limited. However, it is usually done to exclude other neurological conditions that behave like LMS.^6^ Brain MRI showed 1.4 × 1.3 cm low T1 and high T2 right cerebello‐pontomedullary lesion exert homogenous post‐contrast enhancement; findings consistent with LMS. MRI with DWI and MRA are the gold standards to confirm LMS with specificity of 96% and sensitivity of 83% by identifying the exact area of infarction and vascular occlusion.^12^

The goal of treatment is to identify the infarction early, reduce its size, and prevent further complications. The administration of tissue plasminogen activator (TPA) within the first 4.5 h improves the outcome by around 30%.^13^ Our patient presented late, weeks after the onset of the symptoms; thus, administration of TPA was not applicable. Daily antithrombotic therapies with aspirin and/or clopidogrel, along with lipid‐lowering agent statin, reduce the risk for future stroke and improve the outcome in patients with LMS.^14^ Speech therapy is crucial to improve the swallowing function and prevent aspiration. Post‐stroke care with adequate blood pressure and blood glucose control are fundamental. Smoking cessation, regular exercise, healthy diets, and physical therapy are also of extreme importance.

The prognosis of LMS is usually favorable; however, it depends on the size, location, and extent of the infracted area. With appropriate medications and regular care and monitoring, most patients experienced significant improvement, and the majority showed a minimum deficit within 6 months^15^, ^16^ and functional independence within 1 year.^17^

## 5 CONCLUSIONS

Although it is rare, physicians should be familiar with the symptoms and signs of LMS. Urgent neuroimaging is crucial to distinguish LMS from other causes of stroke. The prognosis of LMS is generally good, with the majority experiencing significant improvement within months. Future prospective studies and reported cases in Sudanese culture are needed to better highlight this rare variant of ischemic stroke.

## AUTHOR CONTRIBUTIONS

**Ahmed Alsiddig Ebraheem:** Conceptualization; investigation; writing – original draft; writing – review and editing. **Mumen Abdalazim Dafallah:** Conceptualization; investigation; methodology; writing – original draft; writing – review and editing. **Khawla Abdelmonem Yousef Mohamamed:** Conceptualization; investigation; writing – original draft.

## FUNDING INFORMATION

This research did not receive any specific grant from funding agencies in the public, commercial, or not‐for‐profit sectors.

## CONFLICT OF INTEREST STATEMENT

The authors report no conflicts of interest in relation to the present study.

## ETHICS STATEMENT

Written consent is available in the medical record and with the corresponding author.

## CONSENT

Written informed consent was obtained from the patient to publish this report in accordance with the journal's patient consent policy.

## Data Availability

The data that support the findings of this study are available from the corresponding author upon reasonable request.
